# Death of Alexander III, and His Physicians

**Published:** 1894-12

**Authors:** 


### DEATH OF ALEXANDER III, AND HIS PHYSICIANS.

The death of Alexander III occurred on the 1st instant. It had long been expected, but the exact nature of the disease to which he succumbed is not known in this country, and probably will not be until the official report is received. There has been considerable speculation in this regard. Different physicians have had different opinions, but the accounts have been so meager that this is not to be wondered at.

We are told that he has been, successively, the victim of neurasthenia supervening upon "Russian influenza," of tuberculosis—of the brain, according to one set of physicians; of the kidneys, according to another; of Bright's disease, of cerebral apoplexy, of paresis, of organic disease of the heart, of hereditary insanity, etc.

Aside from the high position which the distinguished patient occupied, more than usual interest has been excited by the reported eccentricities and actions of one of the medical attendants. The account runs:

Sacharjin, or Zacharin, is undoubtedly an eccentric. A native of Moscow, he adheres to the uncouth costume of the Russian peasant, and insisted on visiting the Emperor in a dressing-gown and big boots. On arriving at the palace he refused to occupy the apartments provided for him on the third story, because at home he lived on the ground floor, and required apartments to be provided for him there; he declined to lunch with the Czarina at the imperial table on the ground that he was not in the habit of eating with women; and one day, when the Czarina asked him to visit his patient, whose temperature alarmed her, he replied that he was tired, but would send his assistant; on the return of the latter with a reassuring report Sacharjin turned to the anxious wife and said: "You see I was right not to fuss; there is no danger." The sort of doctor thus portrayed would have suited the first Napoleon, who wanted the Empress to be treated in her accouchement as the wife of the veriest bourgeoise.

Glimpses of this eccentric physician have come down to us from time to time through the dispatches. Here is a paragraph from *The Daily News* correspondent in Vienna, who says: "The gossip about the Czar's doctors threatens to become as painful as in the case of Emperor Frederick. *The Neue Freie Presse's* St. Petersburg correspondent says that while at Spain the Czar was feeling better one night and started playing the trombone. Dr. Zacharin, who was occupying a near-by chamber, sent a letter of request that his Majesty allow him to sleep. The Czar indignantly replied that the Professor need not remain a single night more, but might leave at once if he wished. Zacharin left, and Prof. Leyden was summoned, but as the patient grew worse, Dr. Zacharin was recalled."

Dr. Leyden and Dr. Zacharin did not seem to fraternize, for under date of October 31st we find the following:

Dr. Zacharin and Prof. Leyden have had violent quarrels, each accusing the other of giving the Czar improper treatment. When Prof. Leyden's opinions were accepted Zacharin declared that he would return at once to Moscow. Gen. Ticherevin forbade his leaving Livadia, threatening to detain him by force if necessary.

Of course, at this great distance we cannot appreciate the cause of this disagreement. We only know that human nature is the same the world over, and we recall the violent professional bickerings during the sickness of the German Crown Prince Frederick. Doctors in Russia seem to occupy a peculiar position. —*Medical Examiner for November*.

